# Post-transplant lymphoproliferative disorder after solid organ transplantation: a comprehensive review

**DOI:** 10.3389/frtra.2026.1869288

**Published:** 2026-07-07

**Authors:** Lina Patricia Vargas-Nieto, Nicolás David Santoyo-Sarmiento, Maria Ballesteros-García, Angie Tatiana Calderón-Vásquez, Álvaro Daniel Pinto-Rodriguez, Maria Gabriela Robayo-Romero, Valeria Cormane-Alfaro, Jorge Daza-Buitrago

**Affiliations:** 1 Departamento de Medicina Interna, Fundación Cardioinfantil Instituto de Cardiología, Bogotá, Colombia; 2 Escuela de Medicina y Ciencias de la Salud, Universidad del Rosario, Bogotá, Colombia; 3 Universidad del Rosario, Hospital Universitario Mayor Méderi, Bogotá, Colombia; 4 Grupo de Investigación en Medicina Interna, Corporación Hospitalaria Juan Ciudad - Méderi, Bogotá, Colombia; 5 Facultad de Medicina, Departamento de Oncología, Pontificia Universidad Javeriana, Bogotá, Colombia; 6 Departamento de Hematología, Centro de Tratamiento e Investigación Sobre Cáncer Luis Carlos Sarmiento Angulo, Bogotá, Colombia

**Keywords:** Epstein–Barr virus, histopathological classification, immunosuppression, post-transplant lymphoproliferative disorder, risk stratification, sequential therapy, solid organ transplantation

## Abstract

Post-transplant lymphoproliferative disorder (PTLD) is a serious and heterogeneous neoplastic complication of solid organ transplantation (SOT), arising in the setting of sustained pharmacological immunosuppression. This review is specifically focused on PTLD in the SOT setting; PTLD after hematopoietic stem cell transplantation (HSCT) differs substantially in risk factors, pathogenesis, and management, and is beyond the scope of this work. PTLD incidence ranges from 1% to 20%, depending on the grafted organ, with the highest per-procedure rates in intestinal and multiorgan transplants, and the highest absolute case burden in kidney recipients, given transplant volume. PTLD demonstrates a bimodal temporal distribution: an early, predominantly EBV-driven peak at 12–24 months post-transplant, and a late peak at 5–10 years, with a higher proportion of EBV-negative cases. Contemporary evidence suggests a possible decline in early EBV-positive PTLD with improved surveillance, while late-onset EBV-negative PTLD is stable or increasing. EBV establishes latency type III in PTLD-associated B cells, driving proliferation through viral oncoproteins LMP1 and EBNA2. The latency program correlates with histological category and clinical behavior: latency III predominates in early lesions and polymorphic PTLD with strong EBER expression, whereas EBV-negative monomorphic PTLD displays greater genomic complexity, resembling *de novo*diffuse large B-cell lymphoma (DLBCL), with frequent TP53 mutations and chromosomal gains. The WHO 2022 and ICC 2022 frameworks define four histopathological categories—non-destructive lesions, polymorphic PTLD, monomorphic PTLD, and classic Hodgkin lymphoma (CHL)-type PTLD—each with distinct morphological, immunophenotypic, EBER, and clonality profiles that directly determine treatment intensity. Management follows a sequential strategy: immunosuppression reduction (ISR) as the mainstay first step, followed by rituximab, then chemoimmunotherapy (R-CHOP) for refractory or high-risk disease, with PET/CT-based response assessment using Lugano criteria at each decision point. Tabelecleucel, an allogeneic EBV-specific cytotoxic T-lymphocyte (CTL) product, represents the first approved cellular therapy for refractory EBV-positive PTLD. Immune checkpoint inhibitors carry unacceptably high organ rejection rates and are not recommended for standard PTLD management. Key unmet needs include standardizing EBV surveillance thresholds for preemptive intervention, biomarker-driven risk stratification (PD-L1, LMP1, tumor EBV viral load), and prospective multicenter data on novel immunotherapy combinations in immunosuppressed transplant recipients.

## 1 Introduction

Solid organ transplantation (SOT) constitutes an effective treatment for end-stage organ failure in both pediatric and adult populations, with reported five-year overall survival rates ranging from 39% to 96%, depending on the grafted organ (1). Over the past decade, the number of transplants has increased significantly; in 2023, the United States performed 40,588 transplants, including 21,849 kidney, 9,910 liver, 4,596 heart, 3,016 lung, 917 pancreas, and 95 intestinal transplants (2). The public health impact is substantial, with more than 3.4 million life-years gained between 1987 and 2021, averaging 4.33 years per recipient (3). In liver transplantation, one-year survival exceeds 94%, and median adult life expectancy may reach 20 years (4).

Despite these advances, long-term graft success requires indefinite immunosuppression, commonly comprising calcineurin inhibitors, mTOR inhibitors, thiopurines, and antimetabolites. While indispensable for preventing rejection, these regimens substantially increase the risk of malignancies. Post-transplant lymphoproliferative disorder (PTLD) is among the most clinically serious of these complications, representing approximately 21% of all malignancies in SOT recipients (5–9).

PTLD encompasses a heterogeneous spectrum of lymphoproliferative disorders ranging from reactive, polyclonal early lesions to aggressive monoclonal lymphomas. In pediatric SOT recipients, PTLD accounts for more than 70% of all post-transplant malignancies, with non-Hodgkin lymphoma occurring approximately 21-fold more frequently than in the immunocompetent population (10, 11).

This review focuses specifically on PTLD arising in the SOT setting. PTLD after hematopoietic stem cell transplantation (HSCT) is an important, related, but pathobiologically and clinically distinct entity: it differs from SOT-PTLD in the central role of donor lymphocyte reconstitution kinetics, the impact of T-cell-depleting regimens on disease risk, and the absence of solid allograft rejection as a competing therapeutic risk. Risk factors, surveillance thresholds, and treatment algorithms do not translate directly between these two populations. For HSCT-associated PTLD, readers are directed to dedicated reviews (12, 13). This paper critically analyzes PTLD in modern solid-organ transplantation. It details the disease's epidemiology and clinical presentation, explicitly linking EBV-related and independent pathophysiological mechanisms to their histological categories. Diagnostics are also contextualized using the 2022 WHO and ICC guidelines, ultimately leading to a rigorous evaluation of standard therapies, emerging treatments, and critical gaps in the literature.

## 2 Epidemiology

### 2.1 Incidence, prevalence, and temporal distribution

The incidence of PTLD varies considerably by organ type (65). In adult kidney transplant recipients, the incidence ranges from 0.8% to 2.5%; in liver transplantation, from 1.0% to 5.5%; in heart transplantation, from 2.0% to 8.0%; and in lung transplantation, from 3.0% to 10.0% (9, 14, 15, 67). Intestinal and multiorgan transplants carry the highest per-procedure incidence, reaching up to 20%, attributable to the substantial lymphoid tissue burden of the graft (9, 14). This organ hierarchy reflects the amount of immunologically active donor-associated lymphoid tissue transplanted with the organ, and is a distinct consideration from the overall immunosuppressive load.

Prevalence presents a complementary epidemiological picture. Because kidney transplantation constitutes the majority of all SOT procedures worldwide, kidney recipients account for the largest absolute number of PTLD cases despite their lower per-procedure incidence. The cumulative 10-year prevalence is approximately 3%–4% in kidney and heart recipients (9, 14).

PTLD demonstrates a bimodal temporal distribution: an early peak between 12 and 24 months post-transplant, predominantly EBV-driven, and a late peak between 5 and 10 years, marked by a higher proportion of EBV-negative cases (9, 14, 16). This distribution is not fixed over time. Contemporary series suggest a possible decline in early EBV-positive PTLD, coinciding with improved EBV surveillance and preemptive intervention approaches, while late-onset EBV-negative PTLD is stable or increasing, likely reflecting prolonged immunosuppressive exposure that generates *de novo*lymphomagenesis independent of viral oncogenesis (15, 16). Given these trends, physicians must continue to monitor for PTLD beyond the initial 2-year window, especially in patients on extended calcineurin inhibitor therapy or those who have recently received more intensive immunosuppression.

In the pediatric population, risk is substantially higher than in adults (66). Cumulative two-year incidence reaches 14.1% in EBV-seronegative recipients, with overall prevalence up to 30 times greater than in the age-matched general population (9, 14, 17, 18).

### 2.2 Mortality and survival

Overall survival following PTLD diagnosis remains poor, with outcomes varying by histological subtype, EBV status, and treatment era. Among kidney transplant recipients, reported survival is 72% at six months, 67% at one year, and 54% at three years (13)—figures that compare unfavorably with non-PTLD kidney transplant recipients and underscore the attributable mortality of the disease (68). Adjusted mortality is significantly higher in EBV-seronegative compared with EBV-seropositive recipients, with a hazard ratio of 3.3 (17). PTLD developing within the first three years after heart transplantation is associated with markedly increased early mortality (19). In the relapsed or refractory setting, median overall survival is approximately 4.1 months with anti-CD20-based immunochemotherapy, demonstrating the need for effective salvage options (1).

### 2.3 Risk factors

The most critical individual risk factor for PTLD in the SOT setting is EBV seronegativity in the recipient with a seropositive donor (D+/R−), which increases risk by 10- to 75-fold (17). EBV-associated PTLD in SOT recipients develops predominantly from primary EBV infection in seronegative recipients following transplantation, a setting in which EBV-specific T-cell immunity is absent and pharmacological immunosuppression further impairs viral control. While latent B-cell reactivation can occur in seropositive patients, their existing memory T cells significantly blunt the risk of PTLD. Furthermore, separating this reactivation from a primary infection is nearly impossible if you rely purely on viremia patterns. To tell them apart, clinicians must examine pre-transplant serology and the post-surgical timeline (9, 14).

In addition to EBV status, multiple variables influence PTLD development. The type of transplanted organ is a significant determinant, likely reflecting the graft's lymphoid tissue burden and immunogenicity, with the highest risk observed in intestinal transplants and the lowest in kidney transplants. Patient age is also relevant, as both pediatric and elderly recipients exhibit increased susceptibility. The cumulative intensity of immunosuppression further elevates risk; while antithymocyte globulin induction is a recognized factor, recent lower-dose regimens may be less harmful than previously reported (1, 9, 14). Although certain HLA alleles have been hypothesized to affect EBV immune control, current evidence remains insufficient for clinical application and requires further validation (14).

Pre-transplant rituximab in high-risk recipients and mTOR inhibitor-based maintenance (e.g., sirolimus) have been associated with reduced PTLD risk in selected analyses; however, evidence for rituximab as a broadly applicable PTLD prophylaxis across all SOT types remains limited and should not be generalized beyond specific high-risk subgroups (14, 15, 20). Conversion to an mTOR inhibitor may be considered as part of individualized immunosuppression optimization in patients at elevated risk of PTLD (15). Table 1 summarizes risk factors stratified by onset timing.

**Table 1:** Risk factors for early- and late-onset PTLD in SOT recipients.

Early-onset PTLD (< 12 months)	Late-onset PTLD (≥ 12 months)
EBV D+/R− seromismatch (10- to 75-fold increased risk; primary infection in naive recipient)	Prolonged cumulative immunosuppressive burden and duration
Transplanted organ type: intestinal > lung > heart > liver > kidney (reflects graft-associated lymphoid tissue burden)	Transplanted organ type (lung and multiorgan retain elevated risk)
Induction with polyclonal antilymphocyte antibodies (ATG, OKT3); lower risk with modern lower-dose formulations	Older recipient age at transplantation
Pediatric recipient age (higher background EBV seronegativity)	EBV-negativity (marker of immunosuppression-driven lymphomagenesis, not a causal risk factor *per se*)
Primary EBV infection post-transplant	HLA alleles associated with impaired EBV-specific CTL immune surveillance (limited current evidence)

Adapted from: Allen UD et al. Clin Transplant. 2019;33(9):e13652 (14).; Dierickx D, Habermann TM. N Engl J Med. 2018;378(6):549–562 (9).

### 2.4 Regional epidemiology

Regional PTLD data from Latin America and other low-to middle-income settings are limited, primarily reflecting single-center series with limited generalizability. In Medellín, Colombia, a renal PTLD incidence of 0.47% was reported among 425 kidney transplant recipients from 2005 to 2010 (21), with a subsequent case series demonstrating 87.5% response to ISR combined with mTOR inhibitor conversion and rituximab (22). The predominant histology was monomorphic with 85% EBV association (22). Individual case reports from Bogotá document CNS PTLD and polymorphic PTLD successfully managed with sequential rituximab-CHOP (23, 24). Whether these low rates reflect genuinely reduced regional incidence, underdiagnosis, or reporting patterns is unknown; multicenter prospective registries are needed. Two biological factors may contribute to this regional variability and merit greater attention in the PTLD literature. EBV is not a uniform pathogen: it is divided into type 1 (B95-8 strain) and type 2 (AG876 strain), which differ in the sequences of their EBNA genes, and multiple geographic variants have been characterized over the past decade whose specific disease associations remain incompletely understood (25). Whether strain-specific differences in the efficiency of B-cell immortalization through latency III influence PTLD susceptibility in non-European transplant populations is an open question. The second factor is HLA genetic background. A single-center analysis of 106 PTLD cases and 1,392 transplant controls found that the HLA-B40 group was an independent risk factor in EBV-seronegative recipients (OR 8.38, 95% CI 2.18–32.3), while HLA-B8 conferred risk in EBV-seropositive individuals (OR 3.29, 95% CI 1.52–7.09), and African American race was associated with reduced PTLD risk in the same cohort (26). HLA associations with EBV-positive lymphomas differ between European and non-European populations, and the risk alleles identified in European cohorts do not apply directly to Hispanic or East Asian populations where allele frequencies diverge substantially (27). In Latin America, population data reveal a distinct epidemiological context: EBV-positive lymphomas of T/NK origin are highly prevalent in Mexico and Peru, early EBV seroconversion in childhood occurs throughout the region without the typical mononucleosis syndrome seen in high-income countries, and the burden of EBV-associated malignancy differs markedly from North American and European patterns (28). These data collectively suggest that the PTLD risk profile in Latin American transplant populations may not mirror findings from established registries, reinforcing the need for dedicated regional prospective studies with integrated virological and immunogenetic data.

## 3 Pathophysiology

### 3.1 EBV biology and latency programs

**Figure 1:**
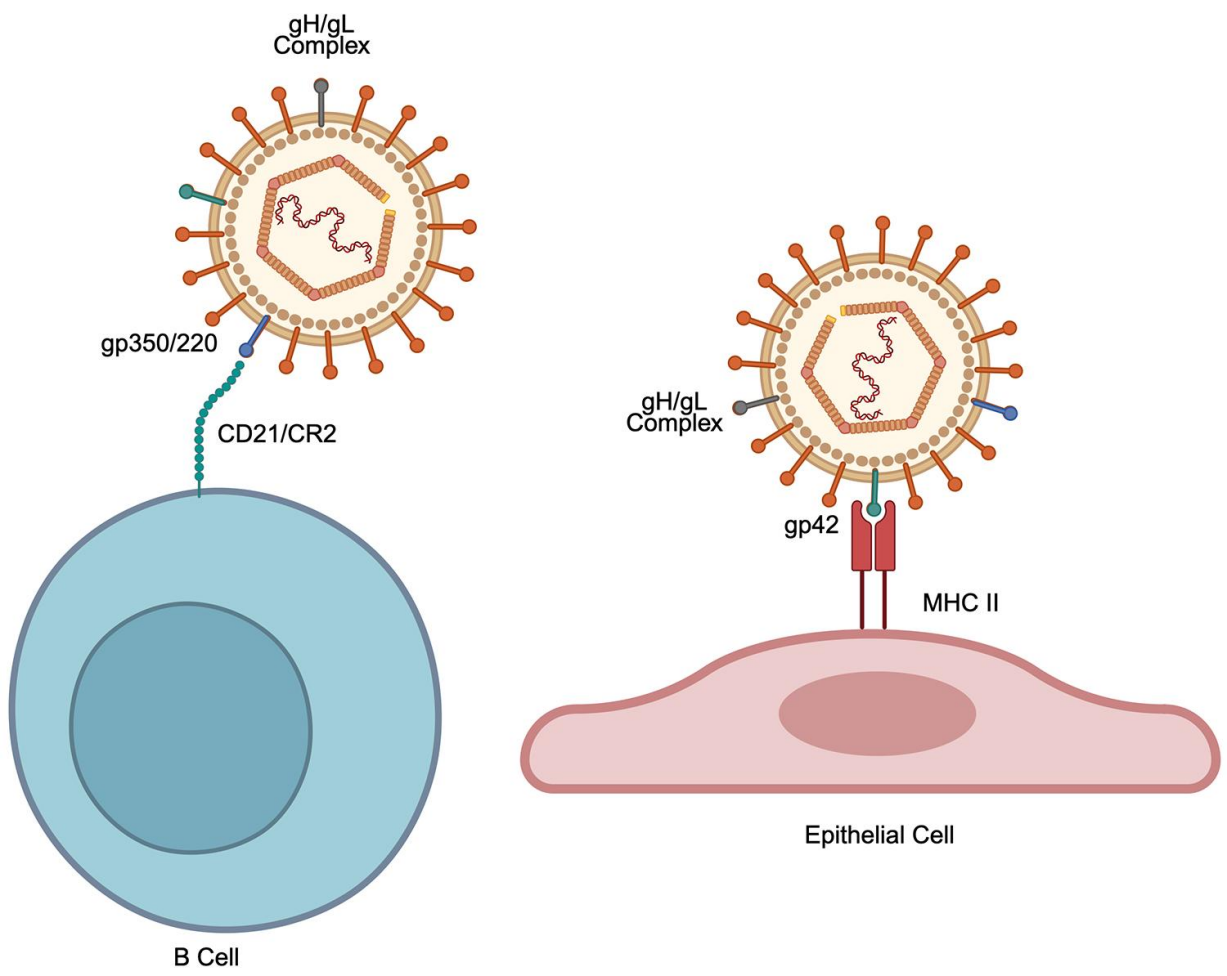
EBV structure and cellular tropism. **(A)** EBV virion architecture showing surface glycoproteins mediating host-cell interactions. **(B)** EBV tropism: in oropharyngeal epithelial cells, gp42 mediates HLA class II-dependent entry; in B cells, gp350/220 engages CD21 (CR2) followed by gH/gL-mediated membrane fusion. EBV, Epstein–Barr virus; gp, glycoprotein; HLA, human leukocyte antigen. Figure produced via BioRender.

Biologically, EBV operates as an enveloped, double-stranded DNA *γ*-herpesvirus. Its 172-kb genome codes for roughly 80 proteins, many of which function as glycoproteins essential for targeting specific cells (Figure 1). Following an initial oropharyngeal infection, the virus generally establishes a lifelong latent state within memory B cells (29, 30).

**Figure 2:**
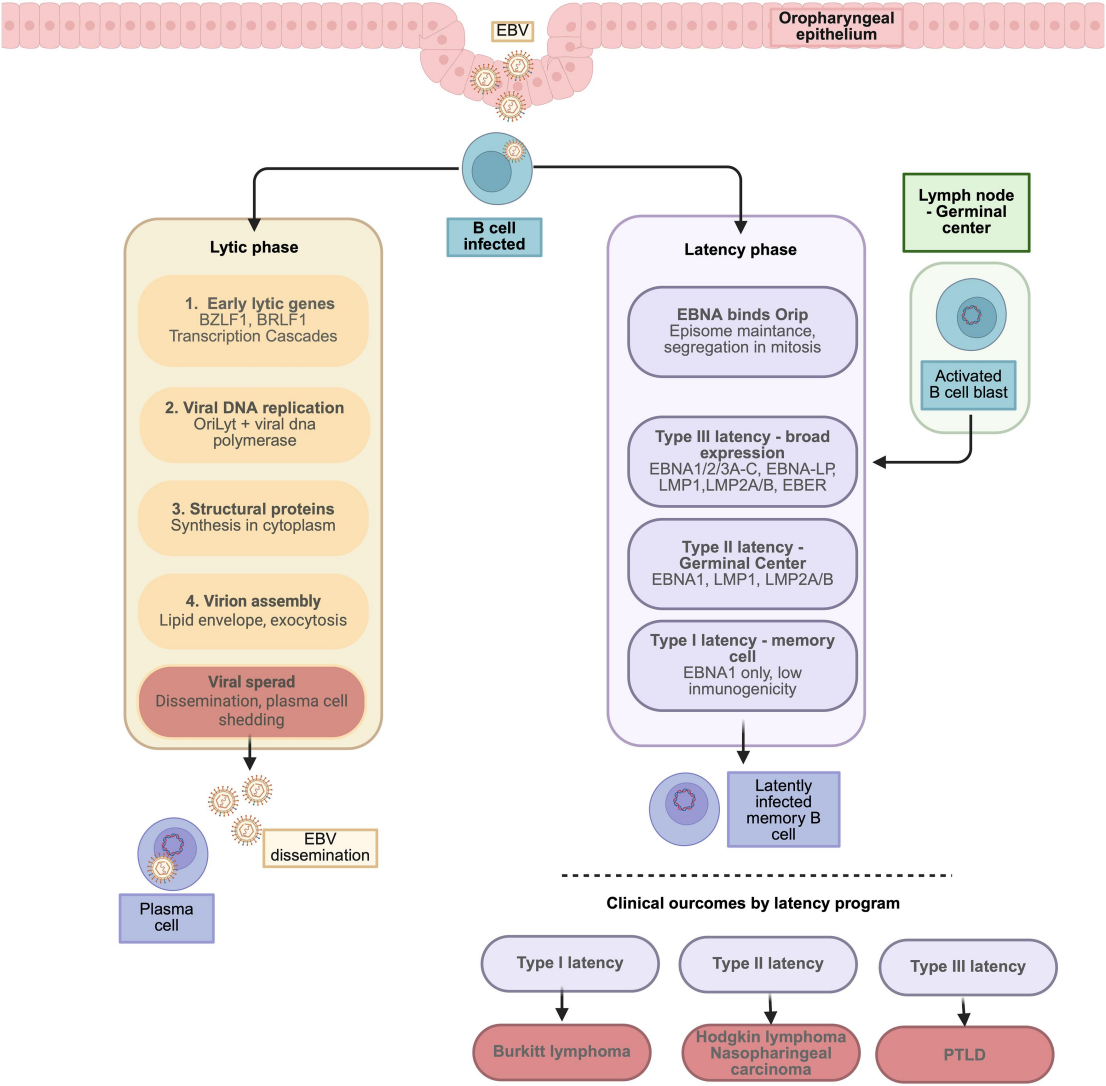
EBV lifecycle and latency programs. Lytic phase: BZLF1/BRLF1-initiated viral DNA replication at OriLyt, virion assembly, exocytic release. Latency phase: EBNA1-mediated episome maintenance via OriP. Latency I (EBNA1 only; Burkitt lymphoma; minimal immunogenicity). Latency II (EBNA1, LMP1, LMP2; Hodgkin lymphoma, nasopharyngeal carcinoma). Latency III (full EBNA/LMP/EBER repertoire; PTLD, lymphoblastoid cell lines; maximal lymphoproliferative and transforming activity; strong EBER expression, LMP1-driven PD-L1 induction). Figure produced via BioRender.

Viral entry into B cells occurs through a sequential series of interactions. Initially, gp350/220 binds to the CD21 (CR2) receptor, followed by gp42 engagement with HLA class II molecules, which facilitates membrane fusion via the gH/gL complex (29). After entry, motor proteins such as dynein and kinesin transport the viral capsid along microtubules to the nucleus (31). Within the nucleus, the linear viral DNA circularizes to form an episome that replicates with the host genome. EBV alternates between lytic and latent phases (Figure 2). During lytic replication, early genes including BZLF1 and BRLF1 initiate viral DNA synthesis, virion assembly, and release. In latency, EBNA1 maintains the episome with minimal gene expression, enabling immune evasion (30, 32).

EBV latency is conventionally classified into three patterns, although there is some biological overlap among them. Latency I, characterized primarily by EBNA1 expression, is typically observed in Burkitt lymphoma and is minimally immunogenic. Latency II includes expression of LMP proteins and is commonly associated with Hodgkin lymphoma and nasopharyngeal carcinoma. Latency III, which involves expression of nearly all EBNAs, LMPs, and EBERs, is the most immunogenic and is most closely linked to the oncogenic processes underlying EBV-driven PTLD (9, 29, 30, 33).

The latency program expressed in PTLD correlates directly with histological category and clinical behavior: early, non-destructive PTLD lesions and polymorphic PTLD predominantly exhibit latency III with strong EBER expression, reflecting highly active EBV-driven lymphoproliferation. Monomorphic PTLD may show latency II or III in EBV-positive cases. Late-onset monomorphic PTLD that is EBV-negative lacks any latency program, reflecting viral-independent lymphomagenesis. In practice, the latency program expressed correlates with morphological category and EBER pattern: strong EBER positivity in a polymorphic lesion is consistent with latency III-driven proliferation, whereas EBER negativity in a large B-cell lymphoma morphology indicates EBV-independent disease and does not exclude PTLD (9, 29, 30).

### 3.2 Viral proteins, oncogenic mechanisms, and biomarker relevance

As the primary EBV oncoprotein, LMP1 acts as a constitutively active mimic of the CD40 receptor (64). This mimicry activates the NF-*κ*B, JAK/STAT, and PI3 K/AKT pathways, driving B-cell survival, proliferation, and the evasion of immune surveillance (34, 35). Additionally, LMP1 upregulates PD-L1 via JAK2-mediated signaling, leading to T-cell exhaustion and facilitating tumor immune escape (35). The detection of LMP1 in biopsy specimens thus yields valuable prognostic information. Clinically, its presence verifies an active latency III program and establishes a biological rationale for PD-1/PD-L1 blockade, even though the severe risk of allograft rejection currently restricts this approach in SOT recipients. Furthermore, LMP1 profiling can help select candidates for EBV-targeted cellular immunotherapies (1, 35).

EBNA1 is essential for episome maintenance and actively inhibits MHC class I-mediated antigen presentation by interfering with proteasome-dependent viral peptide generation, thereby limiting cytotoxic T-cell recognition of infected B cells (36). EBNA2 transactivates both viral (LMP1, LMP2) and cellular proliferation-associated genes (c-Myc, CD21, CD23), while EBNA3A/3C contribute to B-cell immortalization. CTLA-4, a co-inhibitory receptor expressed on activated T cells, interacts with B7-1 (CD80) and B7-2 (CD86) on antigen-presenting cells, reinforcing immunosuppression within the tumor microenvironment (36, 37). Table 2 summarizes the main functions of EBV latent proteins.

**Table 2:** Functions of EBV latent proteins relevant to PTLD pathogenesis.

Protein	Main oncogenic/immunological function	Clinical/biomarker relevance
EBNA1	Episome maintenance via OriP binding. Inhibits MHC class I antigen presentation by blocking proteasomal degradation of viral peptides.	Essential for EBV persistence; target of CTL-based therapies
EBNA2	Viral transactivator of LMP1, LMP2, and cellular genes (c-Myc, CD21, CD23). Master regulator of viral latency III gene expression.	Drives B-cell immortalization in latency III (PTLD)
EBNA3A/3C	B-cell transformation and immortalization. EBNA3B may have tumor suppressor activity. EBNA3A/3C repress tumor suppressors p16 and Bim.	Contribute to cell-cycle dysregulation
EBNA-LP	Cooperates with EBNA2 for B-cell immortalization; required for efficient transcriptional activation.	Latency III marker
LMP1	Principal EBV oncoprotein. CD40 receptor mimic activating NF-*κ*B, JAK/STAT, PI3 K/AKT. Directly induces PD-L1 via JAK2-mediated signaling, enabling T-cell exhaustion.	Potential predictive biomarker; LMP1 + identifies EBV-active disease; target for EBV-CTL; informs checkpoint biology
LMP2A/B	Mimics B-cell receptor signaling; promotes cell survival and inhibits antigen-mediated B-cell activation.	Sustains EBV latency in the absence of antigen stimulation
EBER1/2	Non-coding RNAs. Activate PKR-independent translation, induce IL-10 secretion, and confer resistance to interferon-α-induced apoptosis.	Detected by EBER-ISH (gold standard for EBV status in PTLD tissue); present in latency III; typically negative in EBV-negative PTLD

### 3.3 Immune evasion and the tumor microenvironment

EBV-infected B cells exploit multiple, converging immune evasion mechanisms. EBNA1-mediated inhibition of antigen presentation reduces the efficacy of cytotoxic T lymphocytes (CTLs) (36). EBV promotes IL-10 production, suppressing CTL and antigen-presenting cell activity (35, 69). Downregulation of NKG2D stress ligands limits NK-cell activation (9). LMP1-induced PD-L1 expression engages the PD-1 inhibitory receptor on T cells, promoting immunological tolerance toward EBV-infected B cells (35).

The tumor microenvironment in established PTLD comprises immune cells, cancer-associated fibroblasts, endothelial cells, and extracellular matrix components that continuously interact with transformed B lymphocytes (38). Matrix metalloproteinases and growth factors promote tissue remodeling and tumor migration. Adhesion molecules ICAM-1 and VCAM-1 facilitate malignant cell-endothelium interactions supporting invasion (39, 70). The convergence of PD-L1/PD-1 signaling, CTLA-4 co-inhibition, and IL-10-mediated suppression creates the permissive microenvironment that sustains PTLD progression (35, 38).

Although the rationale for immune checkpoint inhibition in EBV-positive PTLD is biologically compelling, given LMP1-driven PD-L1 overexpression, clinical application is substantially constrained by the risk of allograft rejection. Published series report rejection in approximately 41% and graft loss in 23.5% of SOT recipients receiving immune checkpoint inhibitors (ICIs) (40). Anti-VEGF approaches remain investigational and have no established role in the management of PTLD.

### 3.4 EBV-negative PTLD: genomic and molecular basis

Approximately 30%–50% of PTLD cases arise without detectable EBV infection, and this proportion is increasing with prolonged transplant survival and modern immunosuppressive regimens (1, 39). EBV-negative PTLD displays greater genomic complexity than EBV-positive disease: higher frequency of TP53 mutations; chromosomal gains at 3q, 7q, and 11q24–25; FOXP1 overexpression; and a transcriptomic profile closely resembling *de novo*DLBCL (41, 42). These molecular features reflect a fundamentally different oncogenic pathway: rather than viral protein-driven lymphoproliferation, EBV-negative PTLD develops through accumulation of somatic mutations and genomic instability analogous to immunosuppression-related *de novo*lymphoma.

Several non-mutually exclusive pathogenic mechanisms have been proposed for EBV-negative PTLD. Persistent alloantigen-driven immune activation in the donor graft may sustain chronic B-cell stimulation, ultimately inducing malignant transformation (39). The “hit-and-run” hypothesis posits that an initial EBV infection induces durable epigenetic or genetic alterations that persist after viral clearance, providing a transforming foundation (37). Other herpesviruses, such as CMV, may contribute through chronic antigenic stimulation, though HHV-8 is rarely detected (39). Mechanisms, including microsatellite instability, hypermethylation of tumor suppressor promoters, and aberrant somatic hypermutation, also contribute across both PTLD subtypes (1, 41, 42). EBV-negative PTLD behaves biologically and clinically more like *de novo*DLBCL than EBV-positive polymorphic PTLD, responding poorly to ISR alone and generally requiring rituximab-based chemoimmunotherapy as primary treatment (41, 42). Present the differences between PTLB VEB + and PTLD VEB - in Table 3.

**Table 3:** Comparative features of EBV-positive and EBV-negative PTLD.

Feature	EBV-positive PTLD	EBV-negative PTLD
Typical onset	Early (< 2 years post-transplant)	Late (> 5 years post-transplant)
Age predominance	Younger recipients; pediatric predominance	Older adult recipients
EBV serostatus trigger	Primary infection (D+/R−) or reactivation; D+/R− is the dominant risk scenario	Not EBV-driven; reflects cumulative immunosuppressive burden
Genomic complexity	Low; viral protein-driven lymphoproliferation predominates	High; TP53 mutations, chromosomal gains at 3q, 7q, 11q24-25
Molecular profile	Tolerogenic, immune-evasive phenotype; JAK2-mediated PD-L1/PD-L2 overexpression; LMP1-driven signaling	Resembles *de novo*DLBCL; FOXP1 overexpression; BCL6 rearrangements more common
EBER-ISH	Positive (strong, nuclear); latency III in most cases	Negative
Histological category	All categories; predominant in non-destructive and polymorphic PTLD; also early-onset monomorphic	Predominantly monomorphic PTLD; late-onset; T/NK-cell PTLD frequently EBV-negative
Treatment response to ISR	Higher; up to 77% in polymorphic subtype	Lower; approximately 10% in monomorphic subtype; DLBCL-adapted therapy required
Prognosis	Relatively favorable in non-destructive/polymorphic; poor in R/R monomorphic	Generally poorer; R/R outcomes similar to *de novo*DLBCL
Specific therapeutic options	EBV-specific CTL (tabelecleucel) eligible; rituximab effective for CD20 + disease	DLBCL-adapted chemoimmunotherapy; clinical trial enrollment strongly preferred

## 4 Risk factors

Risk factors for PTLD vary substantially by timing of onset and are summarized in Table 1. Early-onset PTLD (< 12 months post-transplant) is predominantly driven by: (1) EBV D+/R− seromismatch, representing the single highest-magnitude individual risk factor; (2) intestinal or multiorgan transplantation due to graft-associated lymphoid tissue burden; (3) polyclonal antilymphocyte induction therapy, with historical series showing higher risk that may be attenuated with current lower-dose formulations; and (4) pediatric recipient age, reflecting the higher background EBV seronegativity in children (9, 14).

Late-onset PTLD (≥ 12 months) is associated with: cumulative duration and intensity of immunosuppression; lung and multiorgan transplantation, which retain elevated risk; older recipient age; and EBV negativity, which is not a cause *per se* but a marker of immunosuppression-driven, virus-independent lymphomagenesis. This temporal shift reflects an underlying biological transition from EBV-driven lymphoproliferation toward somatic genomic instability accumulated under prolonged pharmacological immunosuppression, analogous to *de novo*lymphoma in other immunodeficiency states (9, 41).

## 5 Clinical presentation

PTLD exhibits a broad clinical spectrum, ranging from asymptomatic incidental findings to fulminant presentations with multiorgan dysfunction (9, 43). Onset is most common within the first year, but late-onset cases occur throughout the transplant follow-up period (9).

Constitutional symptoms are frequent and often nonspecific: persistent fever, night sweats, weight loss, and malaise (43, 44). Lymphadenopathy, adenotonsillar hypertrophy, and cutaneous lesions are commonly observed. Extranodal involvement occurs in more than 50% of cases; common sites include the gastrointestinal tract (abdominal pain, diarrhea, bleeding, perforation), liver (jaundice, elevated hepatic enzymes), kidneys (acute decline in glomerular filtration), lungs (dyspnea, pulmonary infiltrates), and central nervous system (headache, seizures, altered mental status) (19, 38, 43). Bone marrow infiltration may present as unexplained cytopenias (43).

Certain clinical scenarios necessitate immediate tissue biopsy. The first involves pediatric recipients of intestinal or multiorgan transplants with a D+/R− EBV seromismatch who present with rapidly increasing EBV viremia, unexplained fever, and lymphadenopathy; this profile represents the highest risk, and biopsy should not be delayed for spontaneous viremia resolution. The second scenario concerns any transplant recipient who develops new neurological symptoms, such as headache, altered mental status, or seizures. These patients require urgent evaluation for CNS PTLD, including contrast-enhanced MRI and lumbar puncture. Notably, peripheral EBV PCR may yield false-negative results in CNS PTLD, as peripheral viremia can be low or undetectable despite active intracranial disease; thus, a negative blood EBV PCR does not exclude the diagnosis in this context (9, 43). Primary CNS PTLD represents a particularly aggressive subset, occurring in approximately 15%–20% of PTLD presentations and carrying median overall survival below 18 months even with active therapy. An international collaborative analysis of 84 cases identified complete treatment response as the strongest independent predictor of survival (45), and more recent series have highlighted primary CNS PTLD as a distinct neurosurgical entity in which stereotactic biopsy or decompression may be required for tissue diagnosis when lesions cannot be reached by less invasive means (46).

Multiorgan involvement may clinically resemble sepsis or acute allograft rejection, making prompt clinical recognition particularly challenging. In a subset of cases, PTLD resembles infectious mononucleosis, with fever, pharyngitis, cervical lymphadenopathy, myalgias, and arthralgias in the context of EBV reactivation or primary infection (39).

## 6 Diagnostic approach

### 6.1 Clinical suspicion and initial evaluation

Evaluating a patient for PTLD requires a stepwise clinical algorithm rather than a simultaneous panel of tests. The diagnostic workup advances from initial clinical suspicion and peripheral EBV screening directly to imaging-guided biopsy. Once tissue is secured, histological confirmation using the WHO/ICC frameworks dictates the subsequent staging and risk stratification protocols. Consequently, the results of each diagnostic phase strictly determine the next clinical decision (9, 14).

Any SOT recipient presenting with unexplained fever, lymphadenopathy, extranodal mass, organ dysfunction, or rising EBV viremia should be evaluated for PTLD. Initial evaluation includes: comprehensive clinical history and physical examination; complete blood count with differential; serum lactate dehydrogenase (LDH) and uric acid; comprehensive metabolic panel; EBV DNA quantification by PCR in peripheral blood; and pregnancy testing in women of reproductive age (9, 43).

### 6.2 EBV DNA monitoring: value and limitations

EBV PCR quantification in peripheral blood is valuable for surveillance in high-risk patients (D+/R− serology) and for monitoring treatment response in EBV-positive PTLD, but has important limitations. Its positive predictive value for PTLD diagnosis is limited because many immunosuppressed patients maintain chronic EBV viremia without developing malignancy (14). No universal viral load threshold for diagnosis or pre-emptive intervention has been validated; institution-specific protocols therefore vary, and standardization is an unmet research priority (14).

### 6.3 Imaging: staging and biopsy site selection

Imaging serves two complementary roles in PTLD evaluation: defining the anatomical extent of disease to guide treatment planning, and identifying the most accessible and metabolically active site for biopsy. It should be obtained early in the diagnostic process, not reserved for cases in which clinical examination fails to identify an accessible target.

18F-FDG PET/CT is the preferred modality for initial staging, offering sensitivity of 95%–100% and specificity of 80%–95% (33, 43). It enables whole-body disease mapping, identifies the highest-grade lesion for biopsy, and provides the metabolic baseline for subsequent Lugano-criteria response assessment. False-positives occur in infectious or inflammatory conditions; false-negatives are more common in CNS PTLD or T-cell phenotypes (33, 43). Contrast-enhanced CT of the neck, thorax, abdomen, and pelvis is the standard fallback when PET/CT is unavailable. For suspected CNS involvement, contrast-enhanced MRI is the modality of choice with sensitivity exceeding 95% (43). Response to treatment should be formally assessed using the Lugano criteria (complete metabolic response, partial metabolic response, stable disease, or progressive disease) on PET/CT after 2–4 cycles of active therapy, as in standard lymphoma management (14, 47).

### 6.4 Tissue biopsy: technique and diagnostic workup

Securing a reliable diagnosis usually depends on obtaining an adequate tissue biopsy. Excisional biopsy remains the preferred approach because it preserves the underlying tissue architecture. This morphological context is vital for separating early, non-destructive lesions from polymorphic or monomorphic PTLD. However, if an excision poses unacceptable procedural risks, a core needle biopsy offers a reasonable alternative. In contrast, fine-needle aspiration rarely provides enough architectural detail for a comprehensive evaluation.

A standard pathological assessment typically incorporates the following elements (9, 14):

- Histomorphological evaluation using routine hematoxylin and eosin staining.
- An immunohistochemistry (IHC) panel to determine the immunophenotype (incorporating markers such as CD20, CD3, CD10, BCL6, MUM1/IRF4, CD30, CD15, Ki67, and MYC), alongside *κ*/*λ* light chain ISH to assess B-cell lineage.
- EBER *in situ* hybridization (EBER-ISH) to evaluate the presence of latent EBV infection within the tissue.
- Clonality studies evaluating immunoglobulin heavy chain (IgH) gene rearrangements, adding T-cell receptor (TCR) testing if a T-cell lineage is suspected.
- Fluorescence *in situ* hybridization (FISH) targeting *MYC*, *BCL2*, and *BCL6* rearrangements, particularly in monomorphic cases exhibiting a DLBCL-like morphology.

EBER-ISH is mandatory in every PTLD workup to determine EBV status; however, a negative EBER result does not exclude the diagnosis. EBV-negative PTLD is a well-recognized clinicopathological entity, and when the morphological pattern, immunophenotype, and clinical context are consistent with PTLD, the diagnosis should be made regardless of EBER status (41, 42).

Flow cytometry and clonality studies complement morphological assessment by resolving ambiguities that histology alone cannot address. Polytypic *κ*/*λ* light chain expression indicates a polyclonal reactive process, whereas monotypic restriction supports a clonal, neoplastic proliferation. Polyclonal IgH rearrangement by PCR is consistent with a non-destructive or reactive lesion; monoclonal IgH supports PTLD; TCR monoclonality is sought when T-cell PTLD is suspected. These studies are especially useful for distinguishing non-destructive PTLD from reactive hyperplasia and separating polymorphic PTLD from monomorphic B-cell lymphoma (9, 41).

### 6.5 WHO 2022 and ICC 2022 histopathological classification

The current WHO 2022 and ICC 2022 frameworks recognize four diagnostic categories (Table 4), each defined by distinct morphological, immunophenotypic, EBER, and clonality features with direct bearing on prognosis and treatment selection (9, 40, 41):

**Table 4:** WHO and ICC classification of PTLD: evolution and Key diagnostic features.

WHO 2016	WHO 2022	ICC 2022	Morphology & immunophenotype	EBER/clonality/key differential
Plasmacytic hyperplasia PTLD	Plasmacytic hyperplasia (EBV+/−, post-SOT)	Non-destructive PTLD (PH subtype)	Preserved architecture; sheets of mature plasma cells; no destructive infiltration	EBER+; polytypic light chains; polyclonal IgH. Differential: reactive plasmacytosis
Infectious mononucleosis-like PTLD	IM-like PTLD	IM-like PTLD	Paracortical expansion; immunoblasts; T-cell predominance; no architecture destruction	EBER+; polyclonal or polytypic. Differential: acute EBV infection without PTLD
Florid follicular hyperplasia PTLD	Follicular hyperplasia (EBV+/−, post-SOT)	PTLD due to florid follicular hyperplasia	Florid reactive germinal centers; preserved mantle zones and sinuses	EBER variable; polyclonal. Differential: follicular lymphoma
Polymorphic PTLD	Polymorphic LPD (EBV+/−, post-SOT)	Polymorphic PTLD	Destructive growth; heterogeneous infiltrate (small lymphocytes, immunoblasts, plasma cells, centroblasts); residual architecture focally preserved; CD20+, CD30 variable	EBER + (latency III) in majority; oligoclonal/monoclonal IgH. Differential: EBV + DLBCL (distinction by degree of architectural destruction and morphological monotony)
Monomorphic PTLD (DLBCL type)	Lymphoma, EBV+/−, post-SOT	Monomorphic PTLD (DLBCL type)	Sheets of large neoplastic B-cells; GCB or non-GCB (Hans classifier: CD10, BCL6, MUM1); CD20+; CD30 in 70–85%	EBER variable (+ early-onset;−late-onset); consistently monoclonal IgH. Differential: *de novo*DLBCL in transplant recipient (clinical context)
Classic HL PTLD	Classic HL PTLD	Classic HL PTLD	Reed-Sternberg cells in inflammatory background; CD30+, CD15+; CD20 variable/negative; PAX5 dim+	EBER + in ∼80–90%; monoclonal. Differential: nodular LP-PTLD (CD20 + popcorn cells). Managed with ABVD-based CHL protocols

PTLD, post-transplant lymphoproliferative disorder; IM, infectious mononucleosis; LPD, lymphoproliferative disorder; EBV, Epstein–Barr virus; SOT, solid organ transplant; DLBCL, diffuse large B-cell lymphoma; GCB, germinal center B-cell; IgH, immunoglobulin heavy chain; EBER, EBV-encoded RNA; HL, Hodgkin lymphoma; ICC, International Consensus Classification; LP, lymphocyte-predominant; ISR, immunosuppression reduction.

**Non-destructive PTLD** (plasmacytic hyperplasia, infectious mononucleosis-like PTLD, florid follicular hyperplasia): Tissue architecture is preserved. Plasmacytic hyperplasia shows sheets of plasma cells without architectural destruction; infectious mononucleosis-like PTLD shows paracortical expansion with immunoblasts and T-cell predominance mimicking EBV mononucleosis; florid follicular hyperplasia shows markedly expanded reactive germinal centers. Immunophenotypically, all three subtypes demonstrate polytypic immunoglobulin light chains, and EBER is typically positive with a latency III pattern. IgH clonality is polyclonal. Key differential: reactive post-transplant lymphoid hyperplasia without clinical concern. ISR is the primary treatment approach and is associated with high remission rates in this category (9, 41).

**Polymorphic PTLD:** Destructive growth with effacement of underlying architecture by a heterogeneous infiltrate of small lymphocytes, immunoblasts, plasma cells, and centroblasts at various differentiation stages. Residual lymphoid architecture may be focally preserved. Immunophenotypically: CD20+, CD10 variable, BCL6 variable, with EBER-positive latency III pattern in the majority of cases; CD30 expression is variable. IgH shows oligoclonal or monoclonal rearrangements. Critical differential: EBV-positive DLBCL arising in the transplant setting (distinction requires assessment of architecture and degree of morphological monotony). ISR achieves remission in up to 77% of non-destructive and polymorphic cases combined (34).

**Monomorphic PTLD** (the most clinically significant category): This category comprises neoplastic proliferations meeting diagnostic criteria for a recognized lymphoma or plasma cell neoplasm. The most common subtype is diffuse large B-cell lymphoma (DLBCL), characterized by sheets of large neoplastic B cells, followed by Burkitt lymphoma. Plasma cell myeloma, in which the neoplastic population consists of clonal plasma cells rather than lymphoid blasts, is an extremely rare PTLD subtype that, by convention, is assigned to the monomorphic category but has distinct morphological features from lymphoma subtypes. DLBCL-type monomorphic PTLD may be classified as germinal center B-cell (GCB) or activated B-cell (ABC)/non-GCB subtype by the Hans algorithm (CD10, BCL6, MUM1). CD20 is typically positive. EBER status is variable: positive in early post-transplant disease, negative in late-onset cases with high genomic complexity. CD30 is expressed in 70%–85% of cases (40). IgH clonality is consistently monoclonal. Key differentials: *de novo*DLBCL arising coincidentally in a transplant recipient (requires clinical context and timing); EBV-positive DLBCL in other immunosuppressed states. The ISR response rate is approximately 10%, and rituximab-based systemic therapy is required in the vast majority of cases (34).

**Classic Hodgkin Lymphoma (CHL)-type PTLD:** Rare. Classic Reed-Sternberg cell morphology in an appropriate inflammatory background. Immunophenotype: CD30+, CD15+, with loss of pan-B-cell markers (CD20 variable to negative), PAX5 dim positive. EBER is positive in approximately 80%–90% of cases, suggesting a strong association with EBV. Should be managed with CHL-adapted regimens (ABVD-based or BEACOPP) rather than standard PTLD protocols (9, 40). Key differential: nodular lymphocyte-predominant PTLD (LP-PTLD, not included in CHL-PTLD; LPL pattern with CD20 + popcorn cells).

T-cell and NK-cell PTLDs form a distinct and clinically aggressive subset, arising predominantly in an EBV-negative context with late-onset presentation and worse prognosis than B-cell PTLD. Hepatosplenic T-cell lymphoma is the best-recognized subtype in the transplant setting, associated with prolonged azathioprine and anti-TNF exposure. Management follows standard T-cell lymphoma protocols, with allogeneic HSCT consolidation considered in eligible patients (9, 40).

**Figure 3:**
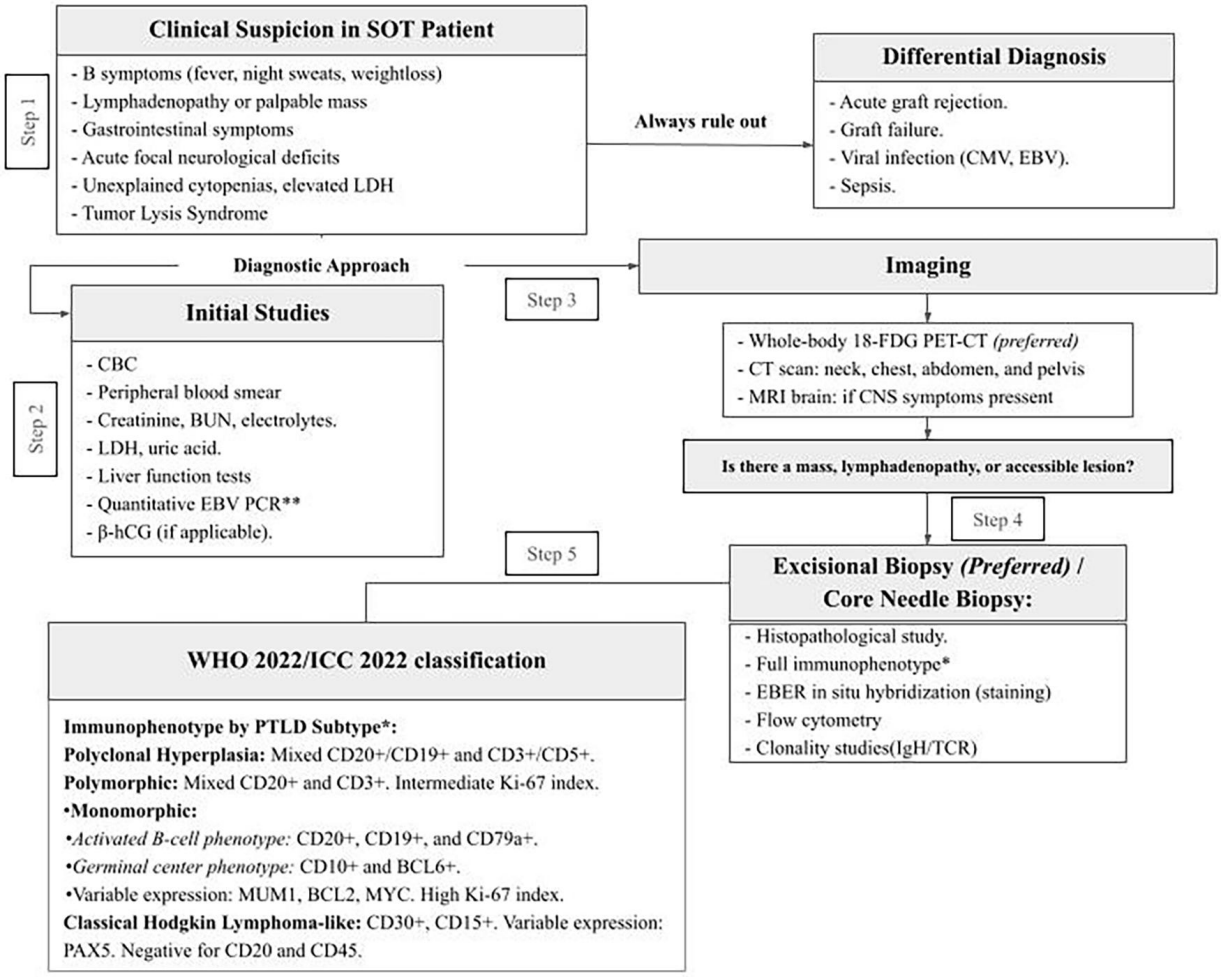
Sequential diagnostic algorithm for PTLD in SOT recipients. Step 1: Clinical suspicion (constitutional symptoms, lymphadenopathy, extranodal mass, unexplained organ dysfunction, or rising EBV viremia). Step 2: EBV DNA quantification by PCR (note: negative peripheral EBV PCR does not exclude CNS or EBV-negative PTLD). Step 3: Imaging — 18F-FDG PET/CT preferred for staging AND biopsy site selection; contrast MRI for CNS. Step 4: Tissue biopsy (excisional preferred; core needle acceptable) with mandatory histopathology, IHC, EBER-ISH, flow cytometry, IgH/TCR clonality, and FISH in monomorphic cases. Step 5: WHO 2022/ICC 2022 classification (non-destructive, polymorphic, monomorphic, CHL-type). PTLD, post-transplant lymphoproliferative disorder; SOT, solid organ transplant; EBV, Epstein–Barr virus; EBER, EBV-encoded RNA; PET/CT, positron emission tomography/CT; IHC, immunohistochemistry; IgH, immunoglobulin heavy chain; TCR, T-cell receptor; ISR, immunosuppression reduction; LP, lumbar puncture; FISH, fluorescence *in situ* hybridization.

During the clinical evaluation, several conditions must be carefully differentiated from PTLD. The primary considerations include acute or chronic allograft rejection, systemic sepsis, and sarcoidosis. It is also important to rule out CMV or EBV infections that lack true lymphoproliferative transformation, as well as incidental *de novo* hematological malignancies. If the initial tissue assessment is inconclusive, PTLD should not be ruled out immediately. In such scenarios, securing a repeat biopsy from a more representative lesion, integrating further molecular assays, or requesting a multidisciplinary pathology review can help clarify the clinical picture before ruling out the disorder (Figure 3) (9, 14, 47).

### 6.6 Staging and pre-treatment stratification

Once the diagnosis and WHO/ICC classification are confirmed, completing a comprehensive staging workup is the next critical step. If not already performed, an 18F-FDG PET/CT is routinely obtained to map disease extent. A bone marrow biopsy is generally reserved for cases in which baseline cytopenias suggest hematological involvement or when the PET/CT reveals suspicious marrow uptake. For patients presenting with neurological signs, the evaluation includes a contrast-enhanced brain MRI and a lumbar puncture to assess CSF cytology and EBV PCR. Finally, before initiating any systemic cytotoxic regimens, standard safety evaluations, including an echocardiogram and baseline viral serologies (HBsAg, anti-HBs, anti-HBc), are strongly recommended (9, 14, 47).

## 7 Management

Treating PTLD requires controlling the malignancy without precipitating graft rejection (48). The standard strategy for B-cell PTLD follows a stepwise progression from immunosuppression reduction (ISR) to rituximab and, if necessary, chemoimmunotherapy (34, 44, 49–51). Treatment intensity is not uniform across cases (34). It is tailored according to histological subtype, EBV status, IPI score, and overall disease stage (44, 50). Patient-specific factors, such as performance status, baseline allograft function, and the risk of acute rejection, also dictate the régimen (34, 44, 48). Managing these overlapping risks relies heavily on continuous dialogue between the transplant team, oncology, and pathology (44).

### 7.1 Immunosuppression reduction

ISR should be considered as the initial management step in most PTLD patients, provided that allograft function and rejection risk permit (44). Standard ISR involves discontinuation of antimetabolites (azathioprine, mycophenolate mofetil) and a 30%–50% reduction in calcineurin inhibitors; conversion to or maintenance of mTOR inhibitors (sirolimus, everolimus) may provide dual benefits of reduced immunosuppression and potential antiproliferative activity (1, 34). In non-destructive and polymorphic PTLD, ISR alone achieves remission in 63%–77% of cases (34, 44). In monomorphic PTLD, ISR remission rates are approximately 10%, necessitating prompt escalation (34). ISR should be individualized in collaboration with the transplant team, with close monitoring for allograft rejection (1). Absence of response within 2–4 weeks, particularly in patients with monomorphic histology, EBV negativity, high tumor burden, or advanced stage, indicates the need for immediate escalation (1, 34).

### 7.2 Rituximab-based therapy

For CD20-positive PTLD refractory to ISR or presenting with high-risk disease, rituximab is the second-line standard (1). Rituximab monotherapy achieves a complete response in 25%–53% of patients, with 10-year overall survival reaching 88% in selected responders (49). The landmark PTLD-1 trial established a sequential regimen of rituximab (four weekly cycles) followed by four cycles of CHOP-based chemotherapy, achieving complete remission in 57% with manageable toxicity; patients with rituximab failure and high IPI were identified as the highest-risk subgroup (50). PET/CT response assessment using Lugano criteria after each therapeutic phase enables objective escalation decisions: patients achieving complete metabolic response after rituximab may avoid chemotherapy; those with partial response or stable disease should proceed to R-CHOP (9, 34, 47, 50).

Situations warranting upfront rituximab plus CHOP-based chemoimmunotherapy include: bulky disease; high IPI (≥ 3); CNS involvement; bone marrow infiltration; and monomorphic EBV-negative PTLD with high genomic complexity (1, 44). The PTLD-2 trial validated subcutaneous rituximab as an effective alternative to intravenous administration within a modified risk-stratified sequential approach in SOT (52).

### 7.3 Refractory and relapsed PTLD

Refractory and Relapse (R/R) PTLD carries a dismal prognosis: median overall survival of approximately 4.1 months with anti-CD20 immunochemotherapy (1, 44). Conventional antiviral therapies have not demonstrated efficacy (1, 34). Platinum-based salvage regimens (R-ICE, R-DHAP) followed by autologous HSCT consolidation are used in eligible patients following R/R DLBCL protocols (1, 34, 44). Despite these approaches, global survival rates in R/R PTLD remain below 20%, and clinical trial enrollment is strongly recommended whenever feasible (1, 44).

### 7.4 Emerging and investigational therapies

Several emerging therapies represent meaningful advances, particularly in the R/R setting, though most data derive from phase I/II studies or retrospective series (34, 40, 53):

**EBV-Specific CTL/Tabelecleucel:** Tabelecleucel is an allogeneic EBV-specific CTL product that targets EBV latency III antigens, representing the first approved cellular immunotherapy for EBV-positive PTLD refractory to rituximab with or without chemotherapy, in SOT or HSCT recipients. Durable responses have been demonstrated in heavily pretreated patients (34, 54). This therapy is currently under access expansion in select regions. It is standard of care for eligible EBV-positive R/R PTLD and should be distinguished from investigational approaches (34, 51).

**CAR-T Cell Therapy:** CD19-directed CAR-T experience in PTLD remains limited. A multicenter retrospective study of 22 post-SOT R/R PTLD patients reported an overall response rate of 64% and a complete response rate of 55%; however, allograft rejection risk and management of cytokine release syndrome in the immunosuppressed transplant context remain major challenges (40).

**Brentuximab Vedotin:** CD30 is expressed in 70%–85% of PTLD cases, making this ADC a relevant option. A phase 1/2 study of brentuximab vedotin plus rituximab reported an overall response of 75% and a complete response of 60% in 20 patients, with grade 3/4 neutropenia in 40% (40).

**Epigenetic Therapy (Nanatinostat):** This histone deacetylase inhibitor enhances the immunogenicity of EBV antigens, potentially synergizing with EBV-specific CTL infusions in EBV-positive PTLD (1).

**Immunomodulatory Agents:** Lenalidomide, an immunomodulatory agent that enhances T and NK cell activity and suppresses angiogenesis, has shown activity in isolated cases of PTLD refractory to conventional therapy. In one report, a patient with T-cell PTLD refractory to multiple chemotherapy regimens achieved complete hematologic normalization with lenalidomide 25 mg/day, sustaining response for nine months before tolerability issues led to dose reduction and discontinuation (55). A second case involved refractory B-cell PTLD in which lenalidomide monotherapy led to complete radiological remission, opening the possibility of high-dose chemotherapy with autologous stem cell support (56). These reports support further investigation, but prospective data in larger cohorts are needed before lenalidomide can be recommended for routine use in any PTLD subtype.

**Immune Checkpoint Inhibitors (ICIs):** The use of ICIs in SOT recipients carries a substantial risk of allograft rejection (reported in approximately 41% of patients) and graft loss (approximately 23.5%), which severely limits their use in this setting (40). Current evidence does not support the routine use of ICIs for PTLD management; their use should be restricted to carefully selected patients with life-threatening, treatment-refractory disease in whom no alternative options remain, with multidisciplinary review and explicit discussion of rejection risk with the patient (40). Although LMP1-driven PD-L1 overexpression in EBV-positive PTLD provides a mechanistic rationale for checkpoint blockade, prospective clinical benefit in SOT recipients has not been established.

**Bortezomib:** Achieves response in ∼50% and complete remission in 20%–30% of EBV-associated R/R PTLD; well tolerated aside from peripheral neuropathy and thrombocytopenia (40).

### 7.5 Rare PTLD subtypes

CHL-type PTLD, T/NK-cell PTLD, primary CNS PTLD, and non-DLBCL B-cell lymphomas require histology-specific management. T/NK-cell PTLD in particular carries a poor prognosis, with few effective options beyond HSCT consolidation; enrollment in clinical trials is particularly encouraged for this subtype (40, 57).

## 8 Prevention and EBV surveillance strategies

Prevention in the SOT setting relies on pre-transplant EBV serological risk stratification, post-transplant EBV DNA monitoring in high-risk recipients, and individualized adjustment of immunosuppressive intensity. No universal viral load threshold for pre-emptive intervention has been established, and monitoring protocols vary substantially between centers; this represents an important gap that prospective trials should address (9, 14, 47). Pre-emptive reduction of immunosuppression or pre-emptive rituximab in EBV-seronegative recipients of high-risk transplants may reduce PTLD incidence in specific populations (particularly pediatric intestinal or multiorgan SOT with D+/R− seromismatch), but should not be applied as a universal prophylaxis across all SOT types without individualized risk-benefit assessment (14, 20, 58, 59).

mTOR inhibitor-based maintenance immunosuppression may reduce PTLD risk in retrospective analyses and can be considered for immunosuppression optimization in high-risk patients at transplant centers experienced in rejection monitoring (15).

Supportive care includes tumor lysis syndrome prophylaxis, Pneumocystis jirovecii pneumonia prophylaxis during intensive therapy; G-CSF support during chemotherapy, and vigilant CNS monitoring in patients with risk features (39, 58, 60).

## 9 Follow-up, recurrence surveillance, and prognostic stratification

Post-treatment surveillance focuses specifically on the risk of PTLD recurrence and long-term allograft preservation (47, 48). Regular clinical assessment should include physical examination, serum LDH, EBV PCR quantification, and PET/CT-based restaging using Lugano criteria at defined intervals, typically every 3 months for the first 2 years, then annually or as clinically indicated (14, 58). The optimal frequency and duration of surveillance remain unstandardized, creating an evidence gap (48).

Reassessment of the immunosuppressive regimen in close consultation with the transplant team is strongly recommended following PTLD remission (47, 48), balancing rejection risk against the potential for immunosuppression-driven PTLD recurrence or EBV reactivation (47, 51). The risk of recurrence is highest in the first 12–24 months after PTLD diagnosis and is substantially elevated in monomorphic and EBV-negative subtypes (44, 48).

### 9.1 Prognostic indices and risk stratification

PTLD prognosis is assessed using validated instruments (49, 51). The International Prognostic Index (IPI) provides a comparable baseline risk stratification for *de novo*DLBCL. PTLD-specific models have been developed and validated in prospective and retrospective cohorts (49–51).

Choquet et al. identified age (≥ 60 years), ECOG PS (≥ 2), elevated LDH, and time since transplant as independent survival predictors, generating a model with one-year survival rates of 100%, 70%, and 36% for low, intermediate, and high-risk categories, respectively (44, 51). Leblond et al. identified PS (≥ 2) and involvement of more than one site as the only independent adverse factors by multivariate analysis in 61 patients (61). Ghobrial et al. confirmed poor functional status, graft involvement, extranodal disease, elevated LDH, and high IPI as adverse determinants in a 107-patient analysis (62). Evens et al. identified CNS involvement, bone marrow involvement, and hypoalbuminemia (the latter not previously described) as independent predictors; three-year PFS was 84%, 66%, and 7% for 0, 1, or ≥2 adverse factors, with rituximab-based first-line therapy significantly improving three-year DFS (70% vs. 21%) and OS (73% vs. 33%) (63).

PD-L1 expression, particularly in LMP1-positive EBV-associated PTLD, tumor EBV viral load, and the mutational landscape of EBV-negative cases are under evaluation as prognostic and predictive markers that may eventually guide treatment selection between EBV-directed cellular therapies and other approaches (34, 35, 39, 50).

## 10 Conclusions and future perspectives

PTLD in solid organ transplant recipients is a complex complication driven by variable biology. While outcomes for aggressive subtypes are often poor, significant gaps in the evidence still limit our understanding. The recent WHO and ICC 2022 frameworks offer a practical foundation for diagnosis and therapy. In everyday practice, clinicians evaluate morphology, immunophenotype, clonality, and the overall clinical context together. Assessing EBV status is routinely required in this workup, though an EBER-negative result does not definitively rule out the disease.

For B-cell PTLD, the standard therapeutic approach typically follows a stepwise escalation from immunosuppression reduction to rituximab, then to chemoimmunotherapy. Treatment decisions along this pathway rely heavily on PET/CT response assessments using the Lugano criteria. The introduction of tabelecleucel presents cellular immunotherapy as a realistic option for refractory EBV-positive cases, and clinical protocols will likely adapt as access broadens. Meanwhile, monomorphic and EBV-negative PTLD pose the greatest challenge, often yielding outcomes comparable to relapsed or refractory DLBCL in an already immunosuppressed host.

Several clinical and research gaps need attention to improve patient care. The field lacks prospective randomized trials to establish clear EBV surveillance thresholds and pre-emptive intervention protocols. There is also a push to individualize treatment intensity based on biomarker profiles, such as PD-L1, LMP1 expression, and genomic complexity. Additionally, evaluating CAR-T cells and checkpoint inhibitors requires multicenter data focused on minimizing allograft rejection. Gathering epidemiological and clinical outcome data through dedicated registries in underrepresented regions, such as Latin America, is equally important. Ultimately, finding ways to trigger an antitumor immune response without sparking alloimmune reactivity is the main biological hurdle for expanding immunotherapy in transplant patients. Overcoming these challenges will depend on active dialogue and shared decision-making across transplant medicine, oncology, and pathology.

## References

[1] Amengual JE Pro B. How I treat posttransplant lymphoproliferative disorder. Blood. (2023) 142(17):1426–37. 10.1182/blood.2023020075 37540819 PMC10731918

[2] Israni AK Zaun DA Martinez A Schaffhausen CR Lozano C McKinney WT. OPTN/SRTR 2023 annual data report: deceased organ donation. Am J Transplant. (2025) 25(2):S490–517. 10.1016/j.ajt.2025.01.026 39947809 PMC12334191

[3] Ferreira LD Goff C Kamepalli S Montgomery AE Miggins JJ Goss JA. Survival benefit of solid-organ transplantation: 10-year update. Dig Dis Sci. (2023) 68(9):3810–7. 10.1007/s10620-023-08012-1 37402977

[4] Lucey MR Furuya KN Foley DP. Liver transplantation. N Engl J Med. (2023) 389(20):1888–900. 10.1056/NEJMra2200923 37966287

[5] Tullius SG Rabb H. Improving the supply and quality of deceased-donor organs for transplantation. N Engl J Med. (2018) 378(20):1920–9. 10.1056/NEJMra1507080 29768153

[6] Stevenson LW. Crisis awaiting heart transplantation. JAMA Intern Med. (2015) 175(8):1406. 10.1001/jamainternmed.2015.2203 26030521

[7] Santarsieri A Rudge JF Amin I Gelson W Parmar J Pettit S. Incidence and outcomes of post-transplant lymphoproliferative disease after 5,365 solid-organ transplants over a 20-year period at two UK transplant centres. Br J Haematol. (2022) 197(3):310–9. 10.1111/bjh.18065 35235680

[8] Christie JD Van Raemdonck D Fisher AJ. Lung transplantation. N Engl J Med. (2024) 391(19):1822–36. 10.1056/NEJMra2401039 39536228

[9] Dierickx D Habermann TM. Post-transplantation lymphoproliferative disorders in adults. N Engl J Med. (2018) 378(6):549–62. 10.1056/NEJMra1702693 29414277

[10] Cheng J Wistinghausen B. Clinicopathologic spectrum of pediatric posttransplant lymphoproliferative diseases following solid organ transplant. Arch Pathol Lab Med. (2024) 148(9):1052–62. 10.5858/arpa.2023-0323-RA 38051286

[11] Robinson C Chanchlani R Kitchlu A. Malignancies after pediatric solid organ transplantation. Pediatr Nephrol. (2021) 36(8):2279–91. 10.1007/s00467-020-04790-2 33057766

[12] Gottschalk S Rooney CM Heslop HE. Post-transplant lymphoproliferative disorders. Annu Rev Med. (2005) 56(1):29–44. 10.1146/annurev.med.56.082103.104727 15660500

[13] Pegoraro F Favre C. Post-transplantation lymphoproliferative disorder after haematopoietic stem cell transplantation. Ann Hematol. (2021) 100(4):865–78. 10.1007/s00277-021-04433-y 33547921

[14] Lee M Abousaud A Harkins RA Marin E Balasubramani D Churnetski MC. Important considerations in the diagnosis and management of post-transplant lymphoproliferative disorder. Curr Oncol Rep. (2023) 25(8):883–95. 10.1007/s11912-023-01418-0 37162742 PMC10390257

[15] Loren AW Porter DL Stadtmauer EA Tsai DE. Post-transplant lymphoproliferative disorder: a review. Bone Marrow Transplant. (2003) 31(3):145–55. 10.1038/sj.bmt.1703806 12621474

[16] Peters AC Akinwumi MS Cervera C Mabilangan C Ghosh S Lai R. The changing epidemiology of posttransplant lymphoproliferative disorder in adult solid organ transplant recipients over 30 years. Transplantation. (2018) 102(9):1553–62. 10.1097/TP.0000000000002146 29485513

[17] Ludvigsen LUP Asberg A Spetalen S Sorensen MD Hamilton-Dutoit S Gramkow AM. Risk and prognosis of posttransplant lymphoproliferative disease in Epstein–Barr virus-seronegative kidney transplant recipients. Am J Transplant. (2025) 25(7):1547–60. 10.1016/j.ajt.2025.01.035 39884653

[18] Sampaio MS Cho YW Shah T Bunnapradist S Hutchinson IV. Impact of Epstein–Barr virus donor and recipient serostatus on the incidence of post-transplant lymphoproliferative disorder in kidney transplant recipients. Nephrol Dial Transplant. (2012) 27(7):2971–9. 10.1093/ndt/gfr769 22273720

[19] Dierickx D Tousseyn T Gheysens O. How I treat posttransplant lymphoproliferative disorders. Blood. (2015) 126(20):2274–83. 10.1182/blood-2015-05-615872 26384356

[20] Walti LN Mugglin C Sidler D Mombelli M Manuel O Hirsch HH. Association of antiviral prophylaxis and rituximab use with posttransplant lymphoproliferative disorders (PTLDs): a nationwide cohort study. Am J Transplant. (2021) 21(8):2532–42. 10.1111/ajt.16423 33289340 PMC8359347

[21] Nieto-Rios J Gomez de los Rios S Serna Higuita L Galvez Cardenas K. Enfermedad linfoproliferativa postrasplante de organo solido. Iatreia. (2016) 29(3):312–322. 10.17533/udea.iatreia.v29n3a06

[22] Nieto-Rios JF Gomez de los Rios SM Serna-Higuita LM Ocampo-Kohn C Aristizabal-Alzate A Galvez-Cardenas KM. Treatment of post-transplantation lymphoproliferative disorders after kidney transplant with rituximab and conversion to m-TOR inhibitor. Colomb Med. (2016) 47(4):196–202. 10.25100/cm.v47i4.1989 PMC5335860 28293043

[23] Acosta Izquierdo L Mora Salazar JA Tramontini C. Sindrome linfoproliferativo postrasplante con compromiso cerebral: reporte de un caso. Rev Chil Radiol. (2016) 22(2):76–9. 10.1016/j.rchira.2016.06.001

[24] Rodriguez Sanchez MP Garcia Padilla PK Contreras KM Gonzalez Gonzalez CA Puentes S. Enfermedad linfoproliferativa en el injerto renal. Reporte de un Caso y Revision de la Literatura. Rev Colomb Nefrol. (2017) 4(2):210. 10.22265/acnef.4.2.218

[25] Neves M Marinho-Dias J Ribeiro J Sousa H. Epstein–Barr virus strains and variations: geographic or disease-specific variants? J Med Virol. (2017) 89(3):373–87. 10.1002/jmv.24633 27430663

[26] Lustberg ME Pelletier RP Porcu P Martin SI Quinion CD Geyer SM. Human leukocyte antigen type and posttransplant lymphoproliferative disorder. Transplantation. (2015) 99(6):1220–5. 10.1097/TP.0000000000000487 25427163

[27] Fletcher LB Veenstra RN Loo EY Hwang AE Siddiqi IN Visser L. HLA Expression and HLA type associations in relation to EBV status in Hispanic Hodgkin lymphoma patients. PLoS One. (2017) 12(3):e0174457. 10.1371/journal.pone.0174457 28334025 PMC5363938

[28] Chabay P Lens D Hassan R Rodriguez Pinilla SM Valvert Gamboa F Rivera I. Lymphotropic viruses EBV, KSHV and HTLV in Latin America: epidemiology and associated malignancies. A literature-based study by the RIAL-CYTED. Cancers (Basel). (2020) 12(8):2166. 10.3390/cancers12082166 32759793 PMC7464376

[29] Young LS Yap LF Murray PG. Epstein–Barr virus: more than 50 years old and still providing surprises. Nat Rev Cancer. (2016) 16(12):789–802. 10.1038/nrc.2016.92 27687982

[30] Murata T Sugimoto A Inagaki T Yanagi Y Watanabe T Sato Y. Molecular basis of Epstein–Barr virus latency establishment and lytic reactivation. Viruses. (2021) 13(12):2344. 10.3390/v13122344 34960613 PMC8706188

[31] Lyman MG Enquist LW. Herpesvirus interactions with the host cytoskeleton. J Virol. (2009) 83(5):2058–66. 10.1128/JVI.01718-08 18842724 PMC2643721

[32] Frappier L. The Epstein–Barr virus EBNA1 protein. Scientifica (Cairo). (2012) 2012:438204. 10.6064/2012/438204 24278697 PMC3820569

[33] Rubinstein J Toner K Gross T Wistinghausen B. Diagnosis and management of post-transplant lymphoproliferative disease following solid organ transplantation in children, adolescents, and young adults. Best Pract Res Clin Haematol. (2023) 36(1):101446. 10.1016/j.beha.2023.101446 36907642

[34] Atallah-Yunes SA Salman O Robertson MJ. Post-transplant lymphoproliferative disorder: update on treatment and novel therapies. Br J Haematol. (2023) 201(3):383–95. 10.1111/bjh.18763 36946218

[35] Taylor GS Long HM Brooks JM Rickinson AB Hislop AD. The immunology of Epstein–Barr virus-induced disease. Annu Rev Immunol. (2015) 33(1):787–821. 10.1146/annurev-immunol-032414-112326 25706097

[36] Levitskaya J Coram M Levitsky V Imreh S Steigerwald-Mullen PM Klein G. Inhibition of antigen processing by the internal repeat region of the Epstein–Barr virus nuclear antigen-1. Nature. (1995) 375(6533):685–8. 10.1038/375685a0 7540727

[37] Rappazzo K Kanakry J Ambinder R. Virus-associated lymphoma. In: Hoffman R Benz E Silberstein L Heslop H Weitz J Anastasi J, editors. Hematology: Basic Principles and Practice. 7th edn. Philadelphia: Elsevier (2018). p. 1439–47.

[38] Markouli M Ullah F Omar N Apostolopoulou A Dhillon P Diamantopoulos P. Recent advances in adult post-transplant lymphoproliferative disorder. Cancers (Basel. (2022) 14(23):5949. 10.3390/cancers14235949 36497432 PMC9740763

[39] Morscio J Tousseyn T. Recent insights in the pathogenesis of post-transplantation lymphoproliferative disorders. World J Transplant. (2016) 6(3):505–16. 10.5500/wjt.v6.i3.505 27683629 PMC5036120

[40] Atallah-Yunes SA Khurana A Habermann TM. Non-DLBCL monomorphic and Hodgkin lymphoma PTLD: clinical insights and treatment strategies. Blood Adv. (2025) 9(22):5792–801. 10.1182/bloodadvances.2025016930 40811832 PMC12662999

[41] El-Mallawany NK Rouce RH. EBV And post-transplant lymphoproliferative disorder: a complex relationship. Hematology Am Soc Hematol Educ Program. (2024) 2024(1):728–35. 10.1182/hematology.2024000583 39644052 PMC11665585

[42] Nakid-Cordero C Choquet S Gauthier N Balegroune N Tarantino N Morel V. Distinct immunopathological mechanisms of EBV-positive and EBV-negative posttransplant lymphoproliferative disorders. Am J Transplant. (2021) 21(8):2846–63. 10.1111/ajt.16547 33621411

[43] Abbas F Kossi ME Shaheen IS Sharma A Halawa A. Post-transplantation lymphoproliferative disorders: current concepts and future therapeutic approaches. World J Transplant. (2020) 10(2):29–46. 10.5500/wjt.v10.i2.29 32226769 PMC7093305

[44] Dharnidharka VR Ruzinova MB Marks LJ. Post-transplant lymphoproliferative disorders. Semin Nephrol. (2024) 44(1):151503. 10.1016/j.semnephrol.2024.151503 38519279 PMC11213680

[45] Cavaliere R Petroni G Lopes MB Schiff D. Primary central nervous system post-transplantation lymphoproliferative disorder: an international primary central nervous system lymphoma collaborative group report. Cancer. (2010) 116(4):863–70. 10.1002/cncr.24834 20052713 PMC4113953

[46] Jin L Lu D Yan F Han J Wei P Zhou Y. A disease warranting attention from neurosurgeons: primary central nervous system post-transplant lymphoproliferative disorder. Front Neurol. (2024) 15:1392691. 10.3389/fneur.2024.1392691 38813246 PMC11133574

[47] Allen UD Preiksaitis JK. Post-transplant lymphoproliferative disorders, Epstein–Barr virus infection, and disease in solid organ transplantation: guidelines from the American Society of Transplantation Infectious Diseases Community of Practice. Clin Transplant. (2019) 33(9):e13652. 10.1111/ctr.13652 31230381

[48] Patil R Prashar R Patel A. Heterogeneous manifestations of posttransplant lymphoma in renal transplant recipients: a case series. Transplant Proc. (2021) 53(5):1519–27. 10.1016/j.transproceed.2021.04.020 34134932

[49] Gonzalez-Barca E Capote FJ Gomez-Codina J Panizo C Salar A Sancho JM. Long-term follow-up of a prospective phase 2 clinical trial of extended treatment with rituximab in patients with B cell post-transplant lymphoproliferative disease and validation in real world patients. Ann Hematol. (2021) 100(4):1023–9. 10.1007/s00277-020-04056-9 32367180

[50] Trappe R Oertel S Leblond V Mollee P Sender M Reinke P. Sequential treatment with rituximab followed by CHOP chemotherapy in adult B-cell post-transplant lymphoproliferative disorder (PTLD): the prospective international multicentre phase 2 PTLD-1 trial. Lancet Oncol. (2012) 13(2):196–206. 10.1016/S1470-2045(11)70300-X 22173060

[51] Choquet S. Treatment of PTLD: a slow and difficult path. Blood. (2024) 144(4):348–50. 10.1182/blood.2024024891 39052268

[52] Zimmermann H Koenecke C Dreyling MH Pott C Duhrsen U Hahn D. Modified risk-stratified sequential treatment (subcutaneous rituximab with or without chemotherapy) in B-cell post-transplant lymphoproliferative disorder (PTLD) after solid organ transplantation (SOT): the prospective multicentre phase II PTLD-2 trial. Leukemia. (2022) 36(10):2468–78. 10.1038/s41375-022-01667-1 35974101 PMC9522585

[53] Daan D Vibeke V. Management of post-transplant lymphoproliferative disorders. HemaSphere. (2019) 3:74–7. 10.1097/HS9.0000000000000226 35309814 PMC8925657

[54] Gupta D Mendonca S Chakraborty S Chatterjee T. Post-transplant lymphoproliferative disorder. Indian J Hematol Blood Transfus. (2020) 36(2):229–37. 10.1007/s12288-019-01182-x 32425371 PMC7229077

[55] Portell C Nand S. Single agent lenalidomide induces a response in refractory T-cell posttransplantation lymphoproliferative disorder. Blood. (2008) 111(8):4416–7. 10.1182/blood-2008-01-132167 18398059

[56] Laeubli H Tzankov A Juskevicius D Degen L Rochlitz C Stenner-Liewen F. Lenalidomide monotherapy leads to a complete remission in refractory B-cell post-transplant lymphoproliferative disorder. Leuk Lymphoma. (2016) 57(4):945–8. 10.3109/10428194.2015.1083563 26295731

[57] Chadburn A. Post-transplant lymphoproliferative disorders (PTLD) in adolescents and young adults: a category in need of definition. Semin Diagn Pathol. (2023) 40(6):401–7. 10.1053/j.semdp.2023.07.002 37596187

[58] Ergisi M Ooi B Salim O Papalois V. Post-transplant lymphoproliferative disorders following kidney transplantation: a literature review with updates on risk factors, prognostic indices, screening strategies, treatment and analysis of donor type. Transplant Rev. (2024) 38(2):100837. 10.1016/j.trre.2024.100837 38430887

[59] Worth A Conyers R Cohen J Jagani M Chiesa R Rao K. Pre-emptive rituximab based on viraemia and T cell reconstitution: a highly effective strategy for the prevention of Epstein–Barr virus-associated lymphoproliferative disease following stem cell transplantation. Br J Haematol. (2011) 155(3):377–85. 10.1111/j.1365-2141.2011.08840.x 21910716

[60] Chiodo Ortiz A Petrossian G Addonizio K Hsiao A Koizumi N Yu Y. Short-term decreased post transplant lymphoproliferative disorder risk after kidney transplantation using two novel regimens. Transpl Immunol. (2023) 76:101774. 10.1016/j.trim.2022.101774 36528248

[61] Leblond V Dhedin N Bruneel MFM Choquet S Hermine O Porcher R. Identification of prognostic factors in 61 patients with posttransplantation lymphoproliferative disorders. J Clin Oncol. (2001) 19(3):772–8. 10.1200/JCO.2001.19.3.772 11157030

[62] Ghobrial IM Habermann TM Maurer MJ Geyer SM Ristow KM Larson TS. Prognostic analysis for survival in adult solid organ transplant recipients with post-transplantation lymphoproliferative disorders. J Clin Oncol. (2005) 23(30):7574–82. 10.1200/JCO.2005.01.0934 16186599

[63] Evens AM David KA Helenowski I Nelson B Kaufman D Kircher SM. Multicenter analysis of 80 solid organ transplantation recipients with post-transplantation lymphoproliferative disease: outcomes and prognostic factors in the modern era. J Clin Oncol. (2010) 28(6):1038–46. 10.1200/JCO.2009.25.4961 20085936 PMC2834429

[64] Furlano PL Bohmig GA Puchhammer-Stockl E Vietzen H. Mechanistic understanding of EBV+ lymphoproliferative disease development after transplantation. Transplantation. (2024) 108(9):1867–81. 10.1097/TP.0000000000004919 39166902

[65] Sprangers B Riella LV Dierickx D. Posttransplant lymphoproliferative disorder following kidney transplantation: a review. Am J Kidney Dis. (2021) 78(2):272–81. 10.1053/j.ajkd.2021.01.015 33774079

[66] Liu Y Sun L Zhu Z Wei L Qu W Wang L. Post-transplant lymphoproliferative disorder after paediatric liver transplantation. Int J Clin Pract. (2021) 75(4):e13843. 10.1111/ijcp.13843 33222369

[67] Aris RM Maia DM Neuringer IP Gott K Kiley S Gertis K. Post-transplantation lymphoproliferative disorder in the Epstein–Barr virus-naive lung transplant recipient. Am J Respir Crit Care Med. (1996) 154(6):1712–7. 10.1164/ajrccm.154.6.8970360 8970360

[68] Morton M Coupes B Roberts SA Klapper PE Byers RJ Vallely PJ. Epidemiology of posttransplantation lymphoproliferative disorder in adult renal transplant recipients. Transplantation. (2013) 95(3):470–8. 10.1097/TP.0b013e318276a237 23222821

[69] Ressing ME van Gent M Gram AM Hooykaas MJG Piersma SJ Wiertz EJHJ. Immune evasion by Epstein–Barr virus. Curr Top Microbiol Immunol. (2015) 391:355–81. 10.1007/978-3-319-22834-1_12 26428381

[70] Tan G Visser L Tan L Berg A Diepstra A. The microenvironment in Epstein–Barr virus-associated malignancies. Pathogens. (2018) 7(2):40. 10.3390/pathogens7020040 29652813 PMC6027429

